# Elephantiasis

**Published:** 1909-12-18

**Authors:** 


### DERMATOLOGY.

### ELEPHANTIASIS.

Elephantiasis is a clinical term which is often used somewhat loosely to indicate any great enlargement of a limb or member or of some part of the body. More strictly speaking, it is employed for such conditions of enlargement as are due to lymph stasis combined with œdema and inflammatory hypertrophy. The well-known elephantiasis of tropical countries, elephantiasis arabum or elephantiasis filariosa, which is associated with filaria sanguinis, is of this class; but it will not be considered here. At present we shall discuss only those cases which come under the head of elephantiasis nostra, and which are of common occurrence in this country. Elephantiasis nostra includes cases of the following types:—(1) Those in which there is enormous enlargement of one lower limb; the leg is cylindrical in shape, with thickened, and often warty or scaly skin, which cannot be pinched up from the deeper tissues. There is some amount of pitting on pressure, but the tissues are very firm and hard, and often strong pressure is required to make any impression. This condition may often be associated with eczema, with varicose ulcer, or with syphilitic ulcers, and there is usually a history of repeated attacks of exacerbations of the swelling with inflammatory symptoms, the meaning of which will be presently explained. (2) Great enlargement of the genital organs—as the penis, or scrotum, or both, or of the labiæ. (3) Thickening of the upper lip or of the eyelids, or of both. As in the case of elephantiasis of the leg it will be found, generally, that there have been repeated erysipelas-like attacks in these parts, and that in some instances these swellings are associated with syphilis or with tuberculosis. Chronic swelling of the upper lip is well known in association with lupus, and when occurring with syphilis it is sometimes so marked that it has received the name of "leontiasis."

This chronic enlargement of limbs or organs is now explained as the result of repeated attacks of cellulitis due to invasion by the streptococcus of erysipelas. It has for long been recognised that repeated attacks of erysipelas may lead to permanent enlargement of the parts affected, and that many cases of elephantiasis are explained in this way, and it is now believed that, in practically all cases of this class, whether associated with syphilitic lesions, with tuberculous lesions, or with simple ulcers and chronic eczema, the cause of the swelling is the same, namely, invasion by the streptococcus. In this way are explained the repeated attacks of inflammatory exacerbation. And it is by the repeated inflammatory attacks that the changes are gradually brought about which lead to the permanent thickening of the skin.

The treatment of these cases consists of first of all attending to any local condition such as eczema, or ulceration, simple, tuberculous or syphilitic. And here it may be remarked that in the syphilitic cases marked diminution of the swelling often takes place with anti-syphilitic remedies. During an inflammatory attack the patient should rest, and the part affected should be kept at rest and fomented. The chronic swelling is to be treated by careful bandaging and by massage, and generally very considerable improvement will take pace. In many of these cases excellent results have been obtained from injections of anti-streptococcic serum.

